# Dual-reactive hydrogels functionalizable using “Huisgen click” and “Schiff base” reactions

**DOI:** 10.55730/1300-0527.3564

**Published:** 2023-04-25

**Authors:** Nergiz CENGİZ

**Affiliations:** 1 Department of Chemistry, Faculty of Arts and Science, Tekirdağ Namık Kemal University, Tekirdağ, Turkey

**Keywords:** Dual-reactive hydrogel, Huisgen click, Schiff base

## Abstract

Hydrogels incorporating different reactive groups are important platforms for the fabrication of functional materials through the conjugation of diverse molecules. In this study, a dual-reactive hydrogel system was designed utilizing aldehyde and azide groups containing methacrylate monomers. Hydrogels were obtained in the presence of a dimethacrylate crosslinker with a combination of hydrophilic PEG-based monomers via free-radical polymerization. The azide and aldehyde sites of the hydrogel network are reactive towards alkyne and amine functional groups, respectively. The advantage of the different reactivities of these functional groups was demonstrated through the attachment of two different dye molecules onto the hydrogel platform via the “Huisgen click” and “Schiff base” reactions to obtain a sensing platform for various applications, such as indicating change in pH of the environment.

## 1. Introduction

Three-dimensional, insoluble macromolecular polymeric structures that have the ability to swell and hold water are defined as hydrogels [1,2]. The design and development of these polymeric networks are crucial since they exhibit many useful properties such as biocompatibility, softness, biodegradability, responsiveness, and reactivity towards various ligands and molecules [3,4]. Due to these versatile functions, hydrogels are widely employed in many biological, environmental, or industrial applications such as drug [5] or protein delivery [6], sensing applications [7–9], contact lenses [10,11], or cell scaffold materials [12,13].

Hydrogels can be synthesized either through covalent [14–16] or noncovalent crosslinking [17] strategies from various types of monomers and polymers. Noncovalent crosslinking strategies include Van der Waals forces, hydrophilic-hydrophobic or ionic interactions, and hydrogen-bonds, though crosslinked networks obtained through these interactions are not always sufficient to make the hydrogel stable. Covalent crosslinking strategies used to design three-dimensional networks comprise covalent bonds which can either be obtained via a thermally-initiated radical polymerization, photopolymerization [18–21], or click-type reactions [22–26].

Actually, “click” reactions, not only enable efficient synthesis of hydrogels but also allow their modification [27] which is of significance in terms of conjugation of molecules of interest to the network. These reactions [28–35] are known to be facile, selective, versatile, and efficient reactions which were first defined by Sharpless [36]. Some of the click reactions that have been applied for the functionalization of polymeric materials are Huisgen 1,3-dipolar cycloaddition [37], Michael addition [38], Diels–Alder [39], radical-mediated thiol-ene [40] and thiol-yne [41], and Schiff base reactions [42]. The conjugations can be carried out through “click-type” reactions among the reactive groups of hydrogels and the complementary reactive groups of the molecule of interest to be attached. Traditional hydrogels which do not contain reactive functional groups are deficient in terms of some application areas, especially enzyme and drug delivery systems or sensing systems. Reactive hydrogels can be an effective remedy for shortcomings of traditional hydrogel systems which limit their applications. For example, Santander-Borrego et al., described the synthesis of PHEMA based hydrogels using HEMA and GMA in the presence of EGDMA crosslinker with a thermal free-radical initiation method [43]. Epoxide groups of the network were reacted with a specific amount of azido alcohol to obtain azido functionalized hydrogel which would be available to achieve covalent immobilization of alkyne-bearing peptide for cellular response. In another study, Becker and colleagues demonstrated the preparation of reactive hydrogels via oxime chemistry by mixing bis-aldehyde functionalized PEG with either a pendant azide or alkene functioanal bis-aminooxy crosslinker. Then, the azide groups were conjugated with alkyne-peptide and alkene units were conjugated with thiol-peptide for tissue engineering applications. Both examples are different in terms of synthesis strategies of hydrogels, but postgelation functionalization is based on “click-type” reactions. In the first example, reactive groups were not affected by the hydrogel synthesis procedures and were available for further modifications [45–47]. In the latter example, the remaining reactive groups after “click-based” crosslinking strategies were used for postmodification. To our surprise, despite many reactive hydrogel examples [48,49], or some dual-reactive polymers [50–52] and polymeric materials [53] in the literature, we did not find dual-reactive hydrogels bearing more than one type of functionality to attach different molecules in an orthogonal fashion. To address this shortcoming, in this study, we exploited monomers that are unreactive towards each other, for the synthesis of dual-reactive hydrogels.

**Figure 1:**
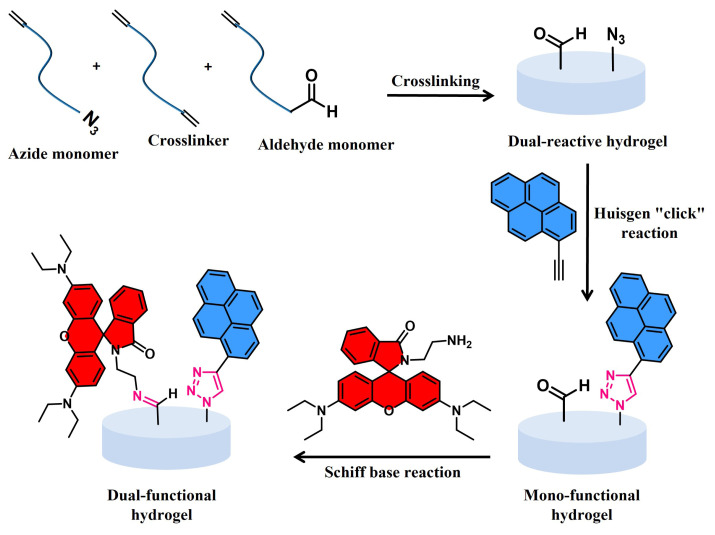
Hydrogel synthesis and dual-functionalization through “Huisgen click” and “Schiff base” reactions respectively.

Herein, utilizing free-radical polymerization technique synthesis of dual-reactive hydrogels from azide- and aldehyde-containing methacrylate monomers, a commercially available PEG-based dimethacrylate crosslinker and a PEG-based hydrophilic monomer is described (Figure 1). To demonstrate the dual-functionalization ability of these hydrogels, the azide group of the hydrogel was first functionalized via “Huisgen click” reaction with 1-ethynylpyrene, a blue fluorescent dye. The blue-fluorescent dye attached hydrogel was then modified with a red fluorescence rhodamine-amine dye using the accessible aldehyde units of the hydrogel network through the “Schiff base” reaction. Rhodamine is well-known to give colorimetric responses to acidic conditions or specific metal ions [54]. For example, Kim et al., synthesized hydrogels incorporating fluorescein and rhodamine moieties to accomplish fluorescence changes in various pH ranges [55]. Recently, we also utilized a metal-catalyst-free amine-epoxy “click” reaction to fabricate hydrogels, and the remaining epoxide groups were functionalized with rhodamine-amine for a colorimetric response. Both pH and metal ion responsiveness were successfully demonstrated for this hydrogel system [26]. Pyrene molecule is another well-known fluorescent sensor. Triazole-linked pyrenyl systems were known to be used for the selectivity of some metal ions, or for exhibiting fluorescence properties [56,57]. Hence, we believe that our dual-reactive hydrogel system can be a good candidate for multi-responsive sensing systems.

## 2. Experimental section

### 2.1. Materials

6-Azidohexyl methacrylate (AHMA) [51], 6-oxohexyl methacrylate (OHMA) [58], and rhodamine-NH_2_ (RHB-NH2) [59] were synthesized as described in literature procedures. Poly(ethylene glycol) methyl ether methacrylate (PEGMEMA, M_n_ = 300 g/mol^−1^), rhodamine B, poly(ethylene glycol) dimethacrylate (PEGDMA, M_n_ = 330 g/mol^−1^), 1-ethynylpyrene, copper(I) bromide (Cu(I)Br), azobisisobutyronitrile (AIBN), potassium hydroxide (KOH), N″, N″-pentamethyldiethylenetriamine (PMDETA), sodium azide (NaN_3_), methacryloyl chloride (MAC), ethylenediamine, and 1,6-hexanediol were obtained from Sigma-Aldrich. 6-chloro-1-hexanol was purchased from TCI. Tetrahydrofuran (THF), methanol (MeOH), dimethyl sulfoxide (DMSO), dimethyl formamide (DMF), dichloromethane (DCM), triethylamine (Et_3_N), and pyridinium chlorochromate (PCC) were obtained from Merck. Prior to use, PEGMEMA and PEGDMA were filtered through a short plug of basic alumina to remove the inhibitors. 1H-NMR spectroscopy characterization of the monomers and dye was conducted by using a Varian 400 MHz instrument. Infrared analyses were carried out using a Bruker Vertex 70 FTIR spectrometer. Fluorescence microscopy images were captured using a Leica CytoVision microscope. Scanning electron microscopy (SEM) images of hydrogels were recorded with QUANTA FEG-250, field emission scanning electron microscope, with low vacuum detector.

### 2.2. Hydrogel synthesis

In a glass vial, AHMA (0.024 g, 0.114 mmol), OHMA (0.021 g, 0.114 mmol) monomers and PEGDMA (0.019 g, 0.057 mmol) crosslinker, were dissolved in MeOH (60 μL) and carefully purged with nitrogen for 15 min at room temperature. AIBN radical initiator (0.005 g, 0.0284 mmol) and MeOH (80 μL) were added to the precursor solution under nitrogen and the reaction mixture was heated at 65 °C for 2 h. The obtained hydrogel (H1) was washed with THF, MeOH, and deionized water, and dried under vacuum (yield 71%). The aforementioned method was also used for the synthesis of hydrogels H2 and H3, which also includes hydrophilic PEGMEMA (M_n_ = 300 g/mol^−1^). The feed monomer ratios (mmol) of AHMA:PEGMEMA:OHMA are 1:0:1, 1:2:1, and 1:6:1 for H1, H2, and H3, respectively as described in Table 1. The ratio of crosslinker in mmol to total monomer in mmol is 0.25 for all procedures.

### 2.3. Water absorption (%) studies of the hydrogels

Hydrogels were first freeze-dried and measured to find initial weights. The samples were placed in deionized water and gently taken out periodically. In every period, the extra surface water was removed using wet filter paper; the mass of the swollen hydrogels was measured and submerged into water. The water absorption amount was measured until the equilibrium swelling. The experiments were conducted in triplicate, and water uptake capacities (%) were calculated using Equation 1 (Eq.1).


Eq.1 $$\%\;Water\;Absorption=\frac{100\times (Wwet-Wdry)}{Wdry}$$

W_wet_: Wet weight of hydrogel, W_dry_: Dry weight of hydrogel

### 2.4. Modification of hydrogel H1 through “Huisgen click” reaction

To the sample of hydrogel H1 (27 mg) in a glass flask, Cu(I)Br (0.34.mg, 0.002 mmol), PMDETA (0.42 mg, 0.002 mmol), and 1-ethynylpyrene (11.00 mg, 0.049 mmol) were added with THF/DMF mixture (1: 0.5 mL) under nitrogen. The mixture was heated at 40 °C for 20 h. Pyrene-functionalized hydrogel (P-H1) was washed multiple times with THF, DMF, and deionized water to eliminate unreacted materials and dried in a vacuum.

### 2.5. Modification of hydrogel P-H1 through “Schiff base” reaction

Rhodamine-NH_2_ (2 mg) was dissolved in DMSO:MeOH solvent mixture (1 mL, 1:1). Pyrene functionalized hydrogel (P-H1, 10 mg) was added to the dye-containing solution and heated at 50 °C for 18 h. Dual-modified hydrogel (PR-H1) was cleaned with DMF, MeOH, and deionized water to eliminate unreacted materials and dried in a vacuum.

## 3. Results and discussion

**Figure 2:**
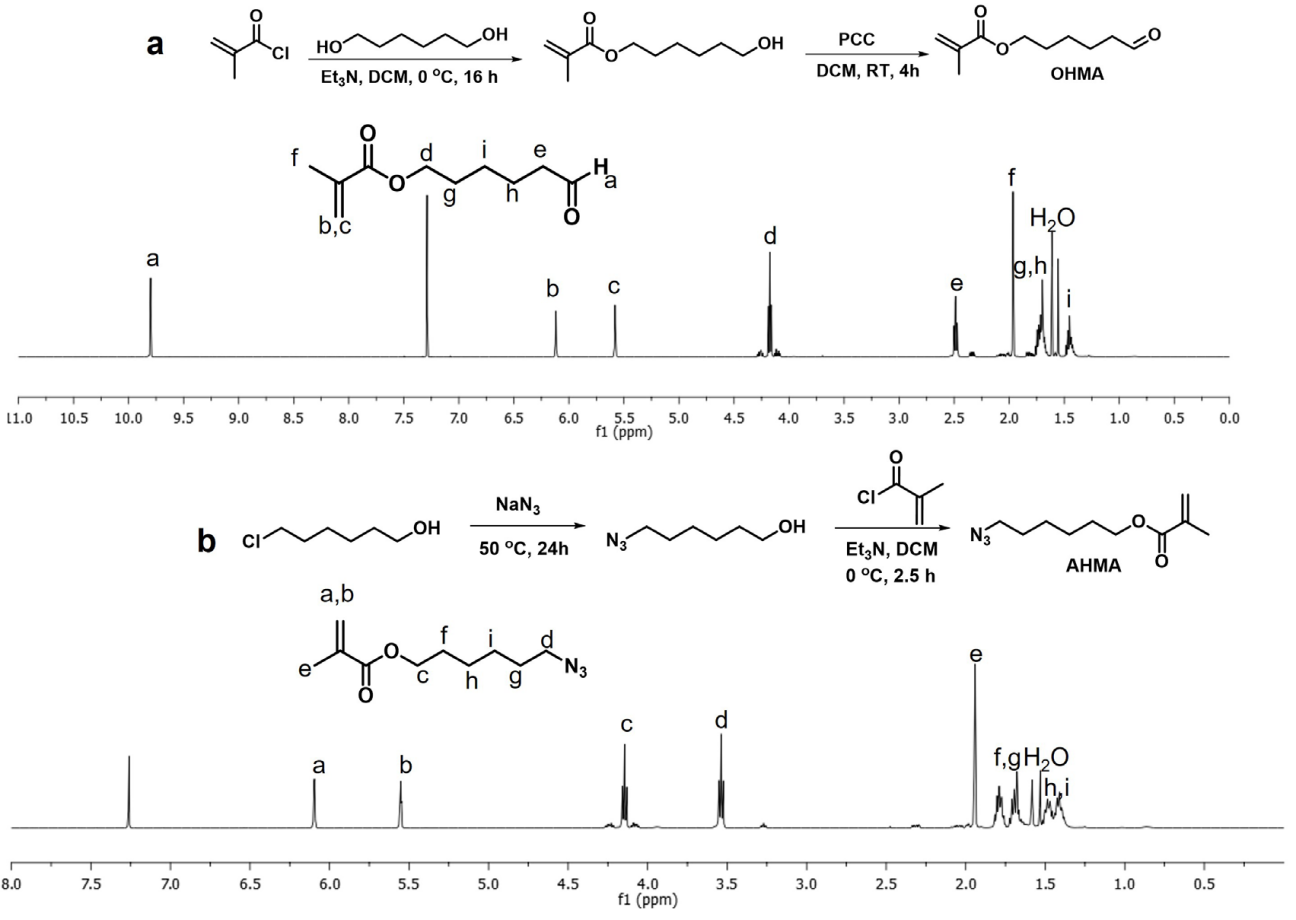
Synthesis procedures and 1H-NMR spectra of OHMA (a) and AHMA (b).

**Figure 3:**
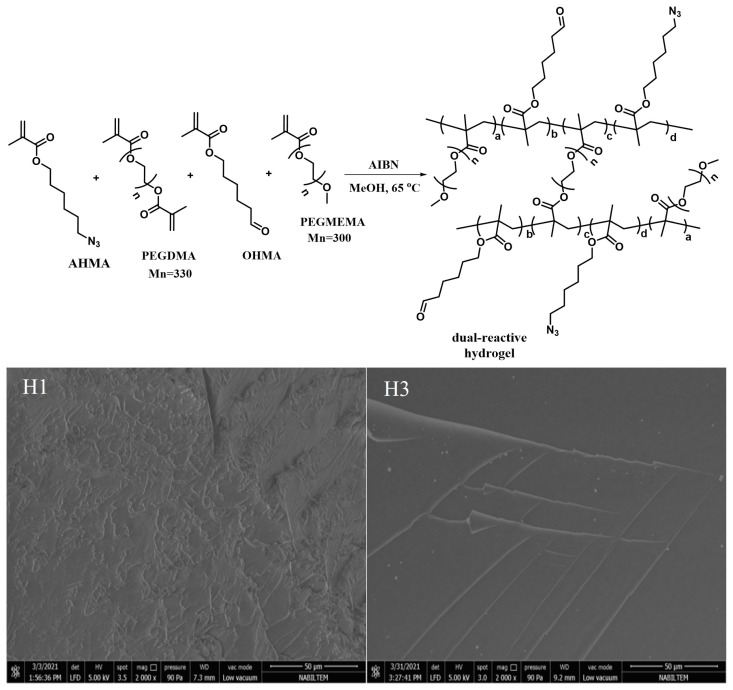
Synthesis of hydrogel network (top), and SEM images of hydrogels H1 and H3 (bottom).

Aldehyde and azide functional groups containing methacrylate monomers OHMA and AHMA were synthesized according to the literature procedures [58,51]. Figure 2 shows the synthesis and 1H-NMR spectra of the monomers. The resonances of the vinylic protons appear at 6.07 and 5.54 ppm, and the aldehyde proton appears at 9.77 ppm for OHMA monomer in Figure 2a. For the AHMA monomer, peaks at 6.09 ppm and 5.55 ppm show the vinylic protons and 3.54 ppm shows the -CH_2_ protons adjacent to azide moiety in Figure 2b. Monomers were also characterized using FTIR spectroscopy, and the azide stretching peak for the AHMA monomer was observed at 2093 cm^−1^. Although no distinct peak for aldehyde was evident in the OHMA monomer, the 1H-NMR spectrum proved its existence beyond doubt. After the monomer synthesis, crosslinking of azide and aldehyde-bearing methacrylate monomers was achieved in the presence of PEGDMA crosslinker in MeOH through AIBN-initiated free radical polymerization at 65 °C, in 2 h to yield (H1). To evaluate the effect of hydrophilic monomer addition to the hydrogel formulation, PEGMEMA was also added to the precursor solution in different ratios (H2, H3), as depicted in Table 1. The molar concentration of PEGDMA crosslinker was adjusted as 25% of the total molar monomer concentration in all gel formulations. Figure 3 (top) shows the representative crosslinking reaction of monomers and crosslinkers through free radical polymerization.

After preparation, hydrogels were swelled in water to reach equilibrium and freeze-dried to analyse their morphologies. SEM images of networks were recorded, and it was observed that none of the hydrogels showed a porous structure (Figure 3, bottom). This nonporous physical image can be the result of both short chain crosslinker PEGDMA (M_n_ = 330 g/mol) usage in the gel and the amount of crosslinker might be relatively high. Table 1 shows the monomer, crosslinker and initiator feed ratios of the hydrogels. For all hydrogel combinations, AHMA/OHMA reactive monomer ratios were kept constant to facilitate comparison. When we increased the amount of PEGMEMA, the yield of the hydrogel reactions was increased up to 92% as seen in Table 1 for hydrogel H3.

**Figure 4:**
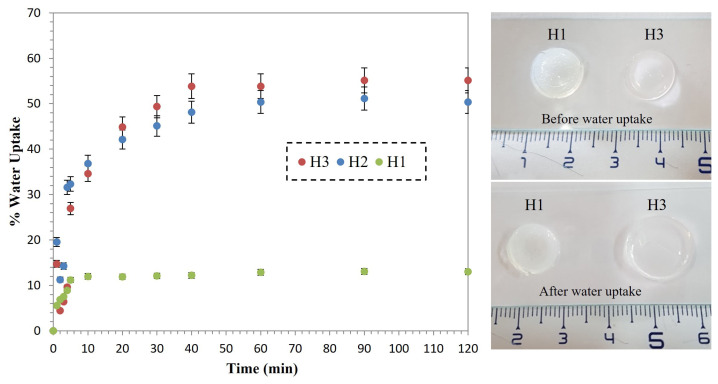
Percentage water uptake versus time graphic of H1, H2, and H3 (left), photographs of H1 and H3 before (right, top), and after swelling in water (right, bottom).

Water uptake capability is an intrinsic feature of hydrogels, where water diffuses inside the network, resulting in expansion of the hydrogel. The swelling behaviour of hydrogels was studied depending on time since this property can be useful for many (bio)applications such as drug delivery. To obtain the swelling profile of hydrogels, the gravimetric method was used immersing preweighed dry hydrogels in aqueous media and removing them at a certain time from the media, and measuring the weight again until the hydrogels reach equilibrium. Then, %water absorption capacity was calculated using Eq. 1. Figure 4 shows the water uptake profile of the hydrogels. As expected, H1 without hydrophilic monomer exhibited the lowest swelling capacity; 13%, while H2 including more PEG units showed 50% capacity, and H3 which has the highest amount of PEG units exhibited 55% water uptake capacity.

**Figure 5:**
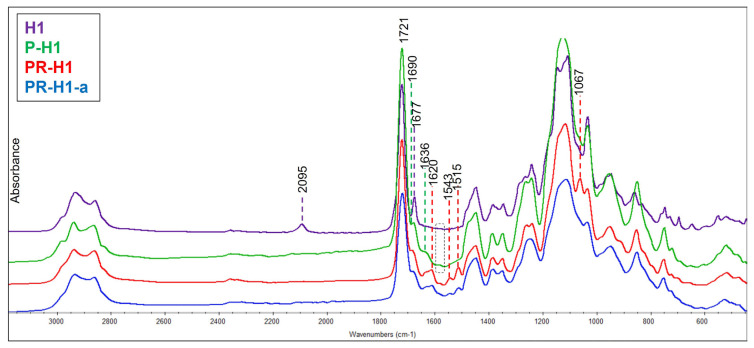
FTIR spectra of hydrogel H1, after functionalization through “Huisgen click” (P-H1), after dual functionalization through the “Schiff Base” reaction (PR-H1), and after treatment with pH 5 acetate buffer (PR-H1-a).

After the synthesis of hydrogels, the feasibility of orthogonal functionalization was demonstrated using two different reactive dye molecules through “Huisgen-type click” reaction and the “Schiff base” reaction. First, azide groups within hydrogel H1 were reacted via the Cu-catalyzed “Huisgen click” reaction in the presence of PMDETA and Cu(I)Br at 40 °C for 20 h with 1-ethynylpyrene which contains a reactive alkyne unit. Figure 5 shows the infrared spectra of the network before and after functionalization through the “Huisgen click” reaction (P-H1). For hydrogel H1, the absorption bands seen at 2095 and 1677 cm^−1^ correspond to the azide and aldehyde groups, respectively. P-H1 spectrum shows the successful attachment of 1-ethynylpyrene through the “Huisgen click” reaction since the azide peaks at 2095 cm^−1^ disappeared completely and new peaks belonging to the aromatic pyrene C=C stretching were observed starting around 1600 cm^−1^ and aromatic C-H bending around 1690 cm^−1^ which overlapped with the aldehyde peak. In addition, the broad peak at 1636 cm^−1^ belonging N=N stretch suggests formation of the triazole moiety [60,61]. Since N=N stretch of triazole ring and aromatic C=C bond stretching due to the pyrene group are expected as a broad peak around the same region, the most crucial evidence for click reaction is the disappearance of azide peak at 2095 cm^−1^.

**Figure 6:**
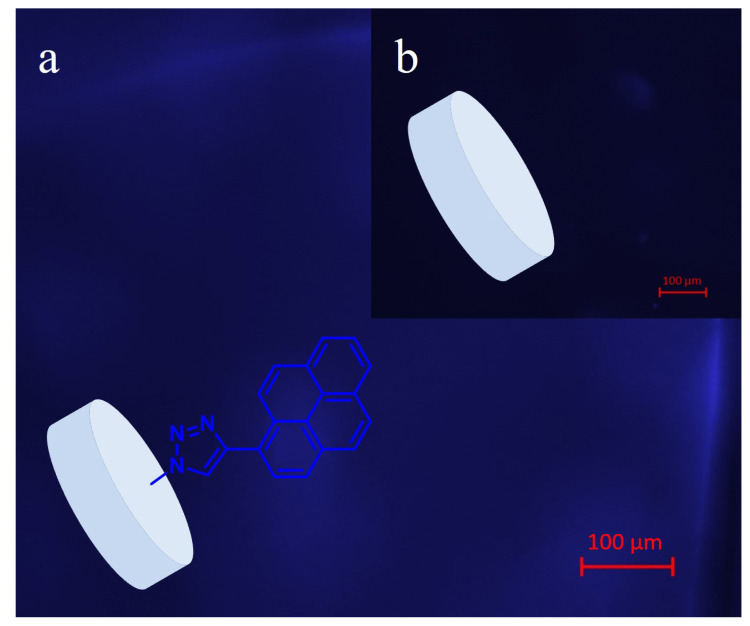
Fluorescence microscopy images of H1 (inset b), and P-H1 upon functionalization with 1-ethynylpyrene (a), Fluorescence images were taken with Hoechst/DAPI (UV); (Excitation BP 350/50, Emission BP 460/50) filter set.

Pyrene attachment to hydrogel H1 was also proved with fluorescence microscopy. The blue fluorescence in Figure 6a results from the pyrene functionality after UV-excitation. The inset image 6b shows the fluorescence microscopy image of hydrogel H1 at the same excitation. As expected, hydrogel H1 which does not incorporate any pyrene group did not show fluorescence.

**Figure 7:**
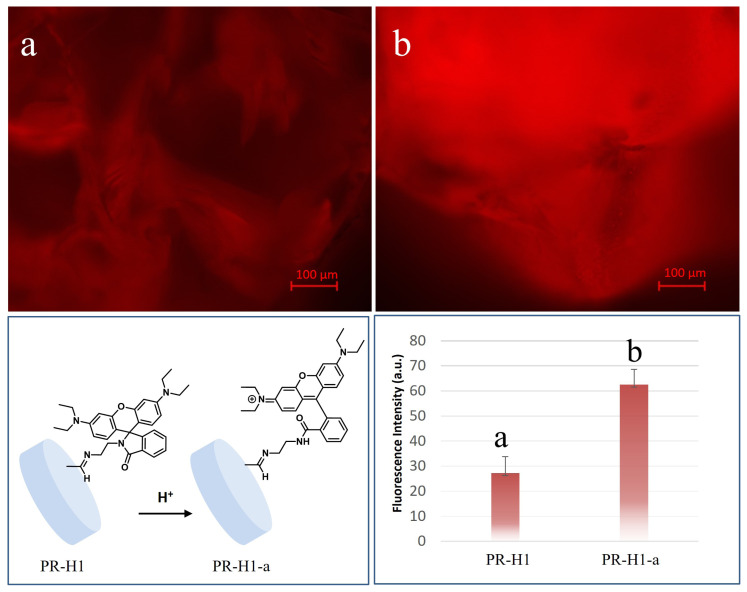
Fluorescence microscopy image of rhodamine functionalized PR-H1 (a, top), and fluorescence image of rhodamine functionalized PR-H1-a after treatment with acid (b, top). Schematic representation of rhodamine group response to acid for PR-H1 (bottom, left). Fluorescence intensity comparison of PR-H1 and PR-H1-a, before and after acid treatment, respectively (bottom, right). Fluorescence images were taken with AF647/CY5(Red); (Excitation BP 620/60, Emission BP 700/75) filter set.

Dual functionalization of hydrogel H1 was shown by further functionalization of P-H1 to obtain PR-H1 through an aldehyde-amine coupling reaction. Hydrogel containing reactive aldehyde groups (P-H1) was reacted with rhodamine-NH_2_ in DMSO:MeOH solvent mixture at 50 °C for 18 h. Then, the characterization of dual-modified hydrogel (PR-H1) was undertaken by FTIR spectroscopy (Figure 5). PR-H1 spectrum exhibits an imine peak at 1620 cm^−1^ which proves the successful functionalization of aldehyde groups with rhodamine-NH_2_ through the Schiff base reaction. Also, characteristic absorption bands of rhodamine due to aromatic C=C stretching can be distinguished between 1600–1500 cm^−1^ [62]. A fluorescence microscopy image was also taken after rhodamine conjugated PR-H1. The red fluorescence in Figure 7a suggests the successful attachment of the rhodamine dye.

It is well-known that the rhodamine group can easily give response to acidic pH [63]. To observe the response, hydrogel PR-H1 was placed in acetate buffer (pH:5, 20 mM) for 5 min and then washed with deionized water and dried under a vacuum. Then, the fluorescence image of acid-treated hydrogel PR-H1-a was found to be considerably brighter compared to the nontreated one, as shown in Figure 7b. The increase in the fluorescence intensity before and after exposure to an acidic environment is quite distinguishable (Figure 7, bottom, right). Depending on the increasing conjugation of rhodamine unit in acidic pH (Figure 7, bottom, left), it is quite reasonable to obtain a higher fluorescence. The FTIR spectrum after acid treatment was also taken, and it was observed that specific rhodamine bands between 1600–1500 cm^−1^ and imine peak at 1620 cm^−1^ survived which proves utility of the system for different applications.

## 4. Conclusion

In conclusion, a dual-functionalizable hydrogel was synthesized using aldehyde and azide bearing reactive monomers through AIBN-initiated free radical polymerization. The incorporation of hydrophilic monomer into the system enhanced the water uptake capacity of the hydrogel system. Facile orthogonal functionalization of the hydrogel with two different dye molecules via the “Huisgen click” reaction and “Schiff base” reaction was accomplished. One can foresee that such orthogonally functionalizable hydrogels can find applications in the fabrication of various sensing platforms.

## Figures and Tables

**Table 1:** Monomer feed ratios and yields of synthesized hydrogels.

	Feed monomer ratio (mmol) AHMA:PEGMEMA:OHMA	AHMA (mmol)	PEGMEMA (mmol)	OHMA (mmol)	PEGDMA (mmol)	AIBN (mmol)	Yield (%)
**H1**	1:0:1	0.114	-	0.114	0.057	0.028	71
**H2**	1:2:1	0.054	0.109	0.054	0.054	0.022	85
**H3**	1:6:1	0.027	0.163	0.027	0.054	0.022	92

Total monomer concentration: 37% WP in MeOH, time: 2 h, Temperature: 65 °C.

%Yield = (W_m_/W_h_) × 100 (W_m_ = Weight of the total monomers and crosslinker, W_h_ = (Weight of the dry hydrogel).

## References

[1] Peppas NA Bures P Leobandung W Ichikawa H Hydrogels in pharmaceutical formulations European Journal of Pharmaceutics and Biopharmaceutics 2000 50 1 27 46 10.1016/S0939-6411(00)00090-4 10840191

[2] Wichterle O Lim D Hydrophilic gels for biological use Nature 1960 185 117 118 10.1038/185117a0

[3] Simic R Mandal J Zhang K Spencer ND Oxygen inhibition of free-radical polymerization is the dominant mechanism behind the “mold effect” on hydrogels Soft Matter 2021 17 26 6394 6403 10.1039/D1SM00395J 34132302 PMC8262556

[4] Ravichandran R Astrand C Patra HK Turner APF Chotteau V Intelligent ECM mimetic injectable scaffolds based on functional collagen building blocks for tissue engineering and biomedical applications RSC Advances 2017 7 21068 21078 10.1039/C7RA02927F

[5] Hoare TR Kohane DS Hydrogels in drug delivery: progress and challenges Polymer 2008 49 8 1993 2007 10.1016/j.polymer.2008.01.027

[6] Kilic Boz R Aydin D Kocak S Golba B Sanyal R Redox-Responsive Hydrogels for Tunable and “On-Demand” Release of Biomacromolecules Bioconjugate Chemistry 2022 33 5 839 847 10.1021/acs.bioconjchem.2c00094 35446015 PMC9121344

[7] Herrmann A Haag R Schedler U Hydrogels and Their Role in Biosensing Applications Advanced Healthcare Materials 2021 10 11 1 25 10.1002/adhm.202100062 PMC11468738 33939333

[8] Buenger D Topuz F Groll J Hydrogels in sensing applications Progress in Polymer Science 2012 37 12 1678 1719 http://dx.doi.org/10.1016%2Fj.progpolymsci.2012.09.001

[9] Sun X Agate S Salem KS Lucia L Pal L Hydrogel-Based Sensor Networks: Compositions, Properties, and Applications—A Review ACS Applied Bio Materials 2021 4 1 140 162 10.1021/acsabm.0c01011 35014280

[10] Kopecek J Hydrogels: From soft contact lenses and implants to self-assembled nanomaterials Journal of Polymer Science Part A: Polymer Chemistry 2009 47 22 5929 5946 10.1002/pola.23607 19918374 PMC2776732

[11] Bettuelli M Trabattoni S Fagnola M Tavazzi S Introzzi L Surface properties and wear performances of siloxane-hydrogel contact lenses Journal of Biomedical Materials Research Part B: Applied Biomaterials 2013 101 8 1585 1593 10.1002/jbm.b.32901 23559566

[12] Gevrek TN Degirmenci A Sanyal R Sanyal A Multifunctional and Transformable ‘Clickable’ Hydrogel Coatings on Titanium Surfaces: From Protein Immobilization to Cellular Attachment Polymers 2020 12 6 1211 10.3390/polym12061211 32466521 PMC7362003

[13] Gevrek TN Cosar M Aydin D Kaga E Arslan M Facile Fabrication of a Modular “Catch and Release” Hydrogel Interface: Harnessing Thiol–Disulfide Exchange for Reversible Protein Capture and Cell Attachment ACS Applied Material & Interfaces 2018 10 17 14399 14409 10.1021/acsami.8b00802 29637775

[14] Hennink WE Nostrum CFv Novel crosslinking methods to design hydrogels Advanced Drug Delivery Reviews 2002 54 1 13 36 10.1016/S0169-409X(01)00240-X 11755704

[15] Patenaude M Smeets NMB Hoare T Designing Injectable, Covalently Cross-Linked Hydrogels for Biomedical Applications Macromolecular Rapid Communications 2014 35 6 598 617 10.1002/marc.201300818 24477984

[16] Kahveci MU Ciftci M Evran S Timur S Yagci Y Photoinduced in situ formation of clickable PEG hydrogels and their antibody conjugation Designed Monomers and Polymers 2015 18 2 129 136 10.1080/15685551.2014.971392

[17] Rosiak JM Yoshii F Hydrogels and their medical applications Nuclear Instruments and Methods in Physics Research Section B 1999 151 1–4 56 64 10.1016/S0168-583X(99)00118-4

[18] Cengiz N Gevrek TN Sanyal R Sanyal A Fabrication of Patterned Hydrogel Interfaces: Exploiting the Maleimide Group as a Dual Purpose Handle for Cross-Linking and Bioconjugation Bioconjugate Chemistry 2020 31 5 1382 1391 10.1021/acs.bioconjchem.0c00108 32259431

[19] Uygun M Kahveci MU Odaci D Timur S Yagci Y Antibacterial Acrylamide Hydrogels Containing Silver Nanoparticles by Simultaneous Photoinduced Free Radical Polymerization and Electron Transfer Processes Macromolecular Chemistry and Physics 2009 210 21 1867 1875 10.1002/macp.200900296

[20] Yilmaz G Kahveci MU Yagci Y A One Pot, One Step Method for the Preparation of Clickable Hydrogels by Photoinitiated Polymerization Macromolecular Rapid Communications 2011 32 23 1906 1909 10.1002/marc.201100470 21910152

[21] Aracier ED Aydin Urucu O Cakmakci E Imidazole modified acrylate-containing photocured hydrogels for the efficient removal of malachite green dye from aqueous solutions Journal of Applied Polymer Science 2021 138 47 51415 10.1002/app.51415

[22] Cengiz N Rao J Sanyal A Khan A Designing functionalizable hydrogels through thiol–epoxy coupling chemistry Chemical Communications 2013 49 95 11191 11193 10.1039/C3CC45859H 24150528

[23] Hwang JH Lee DG Yeo H Rao J Zhu Z Proton Transfer Hydrogels: Versatility and Applications Journal of the American Chemical Society 2018 140 21 6700 6709 10.1021/jacs.8b03514 29767509

[24] Oh J Jung KI Jung HW Khan A A Modular and Practical Synthesis of Zwitterionic Hydrogels through Sequential Amine-Epoxy “Click” Chemistry and N-Alkylation Reaction Polymers 2019 11 9 1491 10.3390/polym11091491 31547408 PMC6780745

[25] Cengiz N Glutathione-responsive multifunctionalizable hydrogels via amine-epoxy “click” chemistry European Polymer Journal 2020 123 109441 10.1016/j.eurpolymj.2019.109441

[26] Cengiz N Fabrication of Multifunctional Stimuli-Responsive Hydrogels Susceptible to both pH and Metal Cation for Visual Detections Macromolecular Chemistry and Physics 2019 220 17 1900212 10.1002/macp.201900212

[27] Yigit S Sanyal R Sanyal A Fabrication and Functionalization of Hydrogels through “Click” Chemistry Chemistry: An Asian Journal 2011 6 10 2648 2659 10.1002/asia.201100440 21954074

[28] Tasdelen MA Diels–Alder “click” reactions: recent applications in polymer and material science Polymer Chemistry 2011 2 10 2133 2145 10.1039/C1PY00041A

[29] Arslan M Acik G Tasdelen MA The emerging applications of click chemistry reactions in the modification of industrial polymers Polymer Chemistry 2019 10 28 3806 3821 10.1039/C9PY00510B

[30] Arslan M Tasdelen MA Click Chemistry in Macromolecular Design: Complex Architectures from Functional Polymers Chemistry Africa 2019 2 195 214 10.1007/s42250-018-0030-8

[31] Bekin Acar S Ozcelik M Uyar T Tasdelen MA Polyhedral oligomeric silsesquioxane-based hybrid networks obtained via thiol-epoxy click chemistry Iranian Polymer Journal 2017 26 6 405 411 10.1007/s13726-017-0529-x

[32] Durmaz H Sanyal A Hizal G Tunca U Double click reaction strategies for polymer conjugation and post-functionalization of polymers Polymer Chemistry 2012 3 4 825 835 10.1039/C1PY00471A

[33] Agar S Baysak E Hizal G Tunca U Durmaz H An emerging post-polymerization modification technique: The promise of thiol-para-fluoro click reaction Journal of Polymer Science Part A: Polymer Chemistry 2018 56 12 1181 1198 10.1002/pola.29004

[34] Gunay US Cetin M Daglar O Hizal G Tunca U Ultrafast and efficient aza- and thiol-Michael reactions on a polyester scaffold with internal electron deficient triple bonds Polymer Chemistry 2018 9 22 3037 3054 10.1039/C8PY00485D

[35] Pektas B Sagdic G Daglar O Luleburgaz S Gunay US Ultrafast synthesis of dialkyne-functionalized polythioether and post-polymerization modification via click chemistry Polymer 2022 253 124989 10.1016/j.polymer.2022.124989

[36] Kolb HC Finn MG Sharpless KB Click Chemistry: Diverse chemical function from a few good reactions Angewandte Chemie International Edition 2001 40 11 2004 2021 10.1002/1521-3773(20010601)40:11<2004::AID-ANIE2004>3.0.CO;2-5 11433435

[37] Kaga S Yapar S Manavoglu Gecici E Sanyal R Photopatternable “Clickable” Hydrogels: “Orthogonal” control over fabrication and functionalization Macromolecules 2015 481 5 5106 5115 10.1021/acs.macromol.5b01536

[38] Arslan M Gevrek TN Sanyal A Sanyal R Cyclodextrin mediated polymer coupling via thiol–maleimide conjugation: facile access to functionalizable hydrogels RSC Advances 2014 4 57834 57841 10.1039/C4RA12408A

[39] Adatia KK Keller S Götz T Tovar GEM Southan A Hydrogels with multiple clickable anchor points: synthesis and characterization of poly(furfuryl glycidyl ether)-block-poly(ethylene glycol) macromonomers Polymer Chemistry 2019 10 4485 4494 10.1039/C9PY00755E

[40] Grim JC Brown TE Aguado BA Chapnick DA Viert AL A Reversible and Repeatable Thiol–Ene Bioconjugation for Dynamic Patterning of Signaling Proteins in Hydrogels ACS Central Science 2018 4 7 909 916 10.1021/acscentsci.8b00325 30062120 PMC6062832

[41] Hu X Tan H Wang X Chen P Surface functionalization of hydrogel by thiol-yne click chemistry for drug delivery Colloids and Surfaces A: Physicochemical and Engineering Aspects 2016 489 297 304 10.1016/j.colsurfa.2015.11.007

[42] Yan S Ren J Jian Y Wang W Yun W Injectable maltodextrin based micelle/hydrogel composites for simvastatin controlled release Biomacromolecules 2018 19 12 4554 4564 10.1021/acs.biomac.8b01234 30350597

[43] Santander-Borrego M Green DW Chirila TV Whittaker AK Blakey I Click functionalization of methacrylate-based hydrogels and their cellular response Journal of Polymer Science Part A: Polymer Chemistry 2014 52 13 1781 1789 10.1002/pola.27183

[44] Lin F Yu J Tang W Zheng J Defante A Peptide-functionalized oxime hydrogels with tunable mechanical properties and gelation behavior Biomacromolecules 2013 14 3749 3758 10.1021/bm401133r 24050500 PMC3871203

[45] Tarakci EC Gevrek TN Isocyanate group containing reactive hydrogels: Facile synthesis and efficient biofunctionalization European Polymer Journal 2022 175 111338 10.1016/j.eurpolymj.2022.111338

[46] Cengiz N Gevrek TN Sanyal R Sanyal A Orthogonal Thiol–Ene ‘Click’ Reactions: A Powerful Combination for Fabrication and Functionalization of Patterned Hydrogels Chemical Communications 2017 53 63 8894 8897 10.1039/C7CC02298K 28740993

[47] Park EJ Gevrek TN Sanyal R Sanyal A Indispensable Platforms for Bioimmobilization: Maleimide-Based Thiol Reactive Hydrogels Bioconjugate Chemistry 2014 25 11 2004 2011 10.1021/bc500375r 25250772

[48] Beria L Gevrek TN Erdog A Sanyal R Pasini D Clickable’ hydrogels for all: facile fabrication and functionalization Biomaterials Science 2014 2 67 75 10.1039/C3BM60171D 32481808

[49] Zou Y Zhang L Yang L Zhu F Ding M “Click” chemistry in polymeric scaffolds: Bioactive materials for tissue engineering Journal of Controlled Release 2018 273 160 179 10.1016/j.jconrel.2018.01.023 29382547

[50] Saha A De S Stuparu MC Khan A Facile and General Preparation of Multifunctional Main-Chain Cationic Polymers through Application of Robust, Efficient, and Orthogonal Click Chemistries Journal of the American Chemical Society 2012 134 41 17291 17297 10.1021/ja307788u 23025462

[51] Cengiz N Kabadayioglu H, Sanyal R Orthogonally functionalizable copolymers based on a novel reactive carbonate monomer Journal of Polymer Science Part A: Polymer Chemistry 2010 48 21 4737 4746 10.1002/pola.24265

[52] Malkoch M Thibault RJ Drockenmuller E Messerschmidt M Voit B Orthogonal Approaches to the Simultaneous and Cascade Functionalization of Macromolecules Using Click Chemistry Journal of the American Chemical Society 2005 127 42 14942 14949 10.1021/ja0549751 16231951

[53] Spruell JM Wolffs M Leibfarth FA Stahl BC Heo J Reactive, Multifunctional Polymer Films through Thermal Cross-linking of Orthogonal Click Groups Journal of the American Chemical Society 2011 133 41 16698 16706 10.1021/ja207635f 21919513

[54] Hu Z-Q Li M Liu M-D Zhuang W-M Li G-K A highly sensitive fluorescent acidic pH probe based on rhodamine B diethyl-2-aminobutenedioate conjugate and its application in living cells Dyes and Pigments 2013 96 1 71 75 10.1016/j.dyepig.2012.07.012

[55] Kim Y Namgung H Lee TS Synthesis of a glucose oxidase-conjugated, polyacrylamide-based fluorescent hydrogel for a reusable, ratiometric glucose sensor Polymer Chemistry 2016 7 6655 6661 10.1039/C6PY01120A

[56] Wu C Ikejiri Y Zhao J-L Jiang X-K Ni X-L A pyrene-functionalized triazole-linked hexahomotrioxacalix[3]arene as a fluorescent chemosensor for Zn2+ ions Sensors and Actuators B: Chemical 2016 228 480 485 10.1016/j.snb.2016.01.051

[57] Atta AK Hazarika SI Loya M Giri S Triazole-linked pyrene appended xylofuranose derivatives for selective detection of Au3+ ions in aqueous medium and DFT calculations 2022 425 113723 10.1016/j.jphotochem.2021.113723

[58] Alconcel SNS Kim SH Tao L Maynard HD Synthesis of Biotinylated Aldehyde Polymers for Biomolecule Conjugation Macromolecular Rapid Communications 2013 34 12 983 989 10.1002/marc.201300205 23553922

[59] Ma C Zeng F Huang L Wu S FRET-Based Ratiometric Detection System for Mercury Ions in Water with Polymeric Particles as Scaffolds The Journal of Physical Chemistry B 2011 115 5 874 882 10.1021/jp109594h 21250732

[60] Pandey P Farha OK Spokoyny AM Mirkin CA Kanatzidis MG A “click-based” porous organic polymer from tetrahedral building blocks Journal of Materials Chemistry 2011 21 1700 1703 10.1039/C0JM03483E

[61] Han NR Cho JW Effect of click coupled hybrids of graphene oxide and thin-walled carbon nanotubes on the mechanical properties of polyurethane nanocomposites Composites Part A: Applied Science and Manufacturing 2018 109 376 381 10.1016/j.compositesa.2018.03.033

[62] Kang W Gao Y Tang X Cao C Hu L Polymer concentration detection method based on fluorescent polymer to evaluate its retention and percolation Journal of Applied Polymer Science 2019 136 19 47468 10.1002/app.47468

[63] Shen S-L Chen X-P Zhang X-F Miao J-Y Zhao B-X A rhodamine B-based lysosomal pH probe Journal of Materials Chemistry B 2015 3 919 925 10.1039/C4TB01763C 32262183

